# Bilingual Processing Mechanisms of Scientific Metaphors and Conventional Metaphors: Evidence *via* a Contrastive Event-Related Potentials Study

**DOI:** 10.3389/fpsyg.2022.894114

**Published:** 2022-06-20

**Authors:** Xuemei Tang, Lexian Shen, Peng Yang, Yanhong Huang, Shaojuan Huang, Min Huang, Wei Ren

**Affiliations:** ^1^School of Foreign Studies, Anhui Polytechnic University, Wuhu, China; ^2^Key Laboratory of Modern Teaching Technology, Ministry of Education, Shaanxi Normal University, Xi’an, China

**Keywords:** scientific metaphors, bilingual, event-related potentials (ERPs), N400, late negativity

## Abstract

To study the different mechanisms of understanding figurative language in a speaker’s native language (L1) and their second language (L2), this study investigated how scientific metaphors in Chinese (L1) and English (L2) are electrophysiologically processed *via* event-related potential experimentation. Compared with the metaphors from daily life or in literary works, scientific metaphors tend to involve both a more complicated context structure and a distinct knowledge-inferencing process. During the N400 time window (300–500 ms), English scientific metaphors elicited more negative N400s than Chinese ones at the parietal region. In the late positive component (LPC) time window (550–800 ms), English scientific metaphors elicited less positive LPCs than Chinese ones at the parietal region, and larger late negativities encompassing smaller areas of the brain. The findings might indicate that for late unbalanced bilingual speakers, L2 scientific metaphor comprehension requires more effort in information retrieval or access to the non-literal route. Altogether, the possible findings are that non-native and non-dominant language processing involves decreased automaticity of cognitive mechanisms, and decreased sensitivity to the levels of conventionality of metaphoric meanings.

## Introduction

The semantic integration between the daily source domain and the scientific target domain involved in scientific metaphor processing provides a perspective for exploring the similarities and differences between processing mechanisms of native language and non-native language (Jankowiak et al., 2017). However, previous studies focused on conventional metaphors and devoted little attention to scientific metaphors. Such metaphors are frequently deployed by scientists who use concrete and intuitive objects and knowledge to describe abstract concepts or objects that are impossible to observe directly. The mechanism of scientific metaphors reflects the core idea of conceptual metaphor theory, the metaphorical understanding of abstract concepts through more specific knowledge structures (Lakoff and Johnson, 1980). The continuous innovation of natural science requires the reorganization and construction of thinking and expression methods in systems related to new concept expression (Tang et al., 2017a,b). Compared with conventional metaphors, the processing of scientific metaphors involves a unique mechanism involving a complicated contextual structure and knowledge inference, which might provide insight in observing the between-language differences of the brain correlates modulated by nativeness, thus deepening the comparative study of bilingual processing mechanisms. Moreover, the comparative study of two different types of metaphors (scientific metaphors and conventional metaphors) might shed new light on the discussion of the processing mechanism of figurative language.

The processing of scientific metaphors differs from that of conventional metaphors mainly in two aspects. First, the contextual structure of scientific metaphors is more complex than that of conventional metaphors, which has been established in previous N400 experiments (Tang et al., 2017a). For example, the source domain (*code*) of the scientific metaphor (*A mitochondrion is a code*) is more frequently used in everyday contexts, while the target domain (*mitochondria*) is more frequently used in scientific contexts. Second, the late processing period of scientific metaphors probably involves a reasoning process from the daily concrete concept to the scientific abstract concept, so as to better comprehend the embedded scientific knowledge. The late components of ERP [the late positive component (LPC) and the late negativity] are very sensitive to this process.

Event-related potentials (ERPs) with extremely high time resolution and rich component dimensions are increasingly used in the study of metaphorical cognitive neural mechanisms. The N400 is considered to be very sensitive to semantic violations (Kutas and Hillyard, 1980), and is an important index to measure the difficulty in retrieving information stored in the mind. Several monolingual studies on figurative meaning processing reported a modulation of N400 amplitudes by the degree of metaphor conventionality (Pynte et al., 1996; Tartter et al., 2002; Coulson and Van Petten, 2007; Lai et al., 2009). Such results seem to be in line with the Career of Metaphor Model (Bowdle and Gentner, 2005), which postulates that novel metaphors are understood through comparison, while conventional metaphors are preferentially analyzed by categorization. Namely, the processing of novel metaphors involves structure alignment between a metaphoric target and a literal base. In contrast, when processing conventional metaphors, the metaphoric target concept is understood as a member of a superordinate category specified by the literal base term (Jankowiak et al., 2017). Thus, understanding novel metaphors requires engagement in sense creation, while understanding conventional metaphors involves easier sense retrieval, as revealed by enhanced N400 amplitudes for novel metaphors (Arzouan et al., 2007a; Lai et al., 2009). In addition to novelty, N400 is also sensitive to concreteness. Forgács et al. (2014, 2015) proposed that most metaphors describe abstract concepts, which, in their study, elicited larger N400s compared with abstract literal expressions when stimulus novelty was controlled across types. In this study, the target words of scientific metaphors were scientific terms and were relatively more abstract than the daily targets of conventional metaphors, which might contribute to the enhanced N400 amplitudes.

The late positive component (LPC) is considered to reflect integration or reprocessing at the sentence level (Kaan et al., 2000). Some monolingual studies found that novel metaphors elicited greater LPCs than conventional metaphors (De Grauwe et al., 2010; Weiland et al., 2014), which indicates that the semantic processes of metaphorical sentences require additional retrieval of information from semantic memory. Other monolingual studies found that novel metaphors elicited smaller LPCs than conventional metaphors (Arzouan et al., 2007a,b; Zhao et al., 2011; Ma et al., 2016), which might be caused by a late negativity overlapping in the LPC time window, reflecting the secondary processing of further semantic integration. Such late negativity is also considered as a continuation of the N400, indicating a sustained difficulty in fusing two concepts from the source domain and the target domain (Goldstein et al., 2012; Rutter et al., 2012).

So far, few studies focused on the processing mechanism of scientific metaphors for bilingual speakers. Thus, expanding a monolingual electrophysiological study on scientific metaphor processing to include a bilingual component might be insightful to enunciate conceptual integration mechanisms when processing semantically complex meanings. Some bilingual ERP studies have reported a lower N400 amplitude elicited by L2 relative to L1 stimuli (Proverbio et al., 2002; Moreno et al., 2008; Midgley et al., 2009; Newman et al., 2012; Heidlmayr et al., 2015), probably indicating weaker interconnectivity for L2 words within the semantic network compared with L1 words (Midgley et al., 2009). Consequently, when processing non-native language, the activity in long-term memory decreases due to weaker interconnectivity. The functional role of the N400 effect is linked to memory operations involved in information retrieval (Kutas and Federmeier, 2000; Kotz et al., 2012). The current study seeks to build on existing research as to semantic processing in bilinguals by using materials with different semantic complexity ranging from highly complex (scientific metaphoric) and relatively complex (conventional metaphoric) to relatively simple (literal) utterances. To minimize the potential influence of any between-group individual differences, a within-subject design was adopted. Accordingly, contrastive analyses of cognitive mechanisms were carried out to investigate native and non-native figurative language comprehension, which can further reveal how semantic complexity and language dominance interact with each other when processing bilingual languages.

The main objective of this study was to observe brain responses to scientific metaphors, conventional metaphors, and literal expressions in Chinese (L1) and English (L2). First, we aimed to observe whether scientific metaphors would evoke higher N400 amplitudes compared with conventional metaphors in both L1 and L2. That is to say, our first focus was the modulation of conventionality for the N400 effect in two languages. A linear N400 effect has been reported by two previous monolingual studies (Tang et al., 2017a,b), with more pronounced N400 amplitudes for scientific metaphors relative to conventional metaphors. This increased processing difficulty was evident due to more demanding mappings required for scientific metaphor comprehension when complicated context and scientific reasoning are engaged. Second, we aimed to observe whether English scientific metaphors would elicit attenuated N400 effects in comparison to Chinese ones, as has been found in previous bilingual research on semantic processing (Weber-Fox and Neville, 1996; Proverbio et al., 2002; Phillips et al., 2004; Moreno and Kutas, 2005; Midgley et al., 2009; Braunstein et al., 2012; Newman et al., 2012; Heidlmayr et al., 2015). Lower N400s elicited by English materials compared with Chinese ones would further imply weaker semantic interconnectivity for L2 when compared with L1 stimuli (Midgley et al., 2009). Third, we aimed to examine whether language nativeness would modulate cognitive mechanisms involved in semantic reintegration at the later stage of processing, as indexed by the LPC response. Several monolingual studies have reported higher LPC amplitudes as the index of secondary semantic integration (van Herten et al., 2005; De Grauwe et al., 2010; Brouwer et al., 2012; Rataj, 2014). It was expected that English scientific metaphors would elicit decreased LPC amplitudes in comparison to Chinese ones. Lower LPCs elicited by English materials relative to Chinese ones would point to weaker semantic processing for L2 compared with L1 sentences (Newman et al., 2012). Furthermore, if the LPC effect is modulated by the conventionality of metaphors (Arzouan et al., 2007a), scientific metaphors should evoke more pronounced LPC amplitudes than conventional metaphors, indicating the continuation of information retrieval or access to the non-literal route. Finally, we expected that main effects between LPCs across items could be observed in both L1 and L2, indicating a similar sensitivity to different levels of conventionality of metaphors.

## Experiment 1 (Chinese L1)

### Method

#### Participants

In total, 20 participants (right-handed, healthy, undergraduate students, L1-Chinese) took part in the ERP experiment. They had either normal or corrected vision and no history of mental illness, neurological disorders, or severe brain damage. All subjects started to learn English from elementary school and have passed the CET-6. All subjects signed a consent and confidentiality agreement before the experiment and received remuneration after the experiment. Finally, the trial data of three subjects were eliminated due to failure to meet the 80% completion-rate threshold. Therefore, the final number of subjects included in our statistical analysis was 17 (7 men, 10 women, average age 21.3 ± 5.32 years).

#### Stimuli

The stimulus pool consisted of 120 sentences, which fell into three categories, namely, scientific metaphors, conventional metaphors, and literal sentences, with 40 sentences in each sentence category. This pool matched the one used in our previous study (Tang et al., 2017a,b) (refer to Table 1 for details).

**TABLE 1:** Sample stimuli.

Scientific metaphors	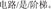	dianlu/shi/jieti.
	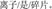	lizi/shi/suipian.
	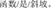	hanshu/shi/xiepo.
	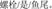	luoshuan/shi/yuwei.
	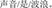	shengyin/shi/bolang.
Conventional metaphors	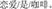	lianai/shi/kafei.
	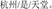	hangzhou/shi/tiantang.
	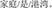	jiating/shi/gangwan.
	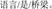	shouji/shi/huoban.
	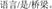	yuyan/shi/qiaoliang.
Literal expressions	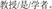	jiaoshou/shi/xuezhe.
	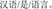	hanyu/shi/yuyan.
	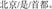	beijing/shi/shoudu.
	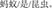	mayi/shi/kunchong.
	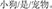	xiaogou/shi/chongwu.

#### Procedure

**FIGURE 1:**
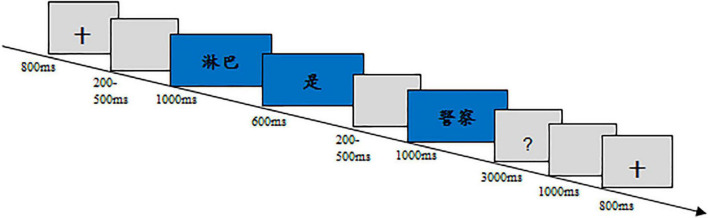
The procedure of Experiment 1.

The experiment took place in a sound-attenuated, electrically shielded room. Participants were seated 80 cm away from the display screen. The sentences were presented in white color on a black background, word by word in a quasi-random order. As illustrated in Figure 1, stimuli in each trial were presented in the following time sequence: fixation cross (800 ms), blank (200–500 ms), subject (1,000 ms), verb (600 ms), blank (200–500 ms), object (1,000 ms), and question mark (3,000 ms). At the sight of the question mark, participants were asked to do a semantic judgment by pressing a corresponding key with right and left index fingers. The whole experiment consisted of four blocks interspersed with three rest intervals. To familiarize the subjects with trial procedure and operation, a practice session was done before the formal experiment. To mitigate any difficulty in understanding the scientific terms involved in the stimuli, participants were first asked to read a list covering all the scientific terms used.

#### EEG Recording and Data Analysis

Scalp voltages were collected using the CURRY 7 system (Compumedics Neuroscan, Texas, United States) with 64 Ag/AgCl electrodes, monitored using the CURRY recording software and connected to a SynAmp amplifier (Compumedics Neuroscan, Texas, United States). Amplified analog voltages were digitized at 1,000 Hz. Impedances of individual sensors were kept below 5 kΩ. Eye movements were monitored through bipolar electrodes, which were placed above and below the right eye, as well as at the left and right canthi. Electroencephalography (EEG) was measured online with reference to the left mastoid, with a ground electrode on the medial frontal aspect, and later was analyzed offline with re-reference to an average of the left and right mastoids.

EEG was analyzed using the SCAN 4.5 software (Compumedics Neuroscan, Texas, United States) and Matlab using the ERPLAB toolbox (Lopez-Calderon and Luck, 2014). The EEG was digitally filtered at 0.1–30 Hz bandpass. Eye movements were corrected with an ocular artifact correction algorithm (Gratton et al., 1983). Artifacts with amplitudes exceeding ±75 μV were removed from analyses. ERPs were time-locked to the onset of the last word of the sentence and were obtained by stimulus-locked averaging of the EEG recorded in each condition. Epochs were 1,000 ms in length with a 200 ms pre-stimulus baseline. The resulting amplitudes of N400 and LPC were entered into 3 condition × 3 region (frontal F3, Fz, F4, central C3, Cz, C4, parietal P3, Pz, P4) × 3 hemisphere (left F3, C3, P3, midline Fz, Cz, Pz, right F4, C4, P4) three-way ANOVAs for repeated measures. All ANOVA results were Greenhouse–Geisser corrected if assumption of sphericity was violated, and *post-hoc* multiple comparisons were carried out using Bonferroni-adjusted corrections.

### Results

#### Behavioral Results

A repeated-measures ANOVA revealed significant effects of condition for reaction time *F*(2,32) = 5.32, *p* = 0.01, *η_*p*_^2^* = 0.25. Pairwise comparisons showed that the reaction time of scientific metaphors was significantly longer than that of literal sentences (*p* = 0.005). There was neither significant difference between conventional metaphors and literal sentences nor between the two metaphorical conditions (*p*-values > 0.1). For accuracy rates, a main effect between conditions was found [*F*(2,32) = 31.12, *p* < 0.001, *η_*p*_^2^* = 0.49]. Pairwise comparisons showed that the accuracy rate of scientific metaphors was significantly lower than that of conventional metaphors and literal sentences (*p*-values < 0.01). There was no significant difference between conventional metaphors and literal sentences (*p* = 0.569).

#### Electrophysiological Results

**FIGURE 2:**
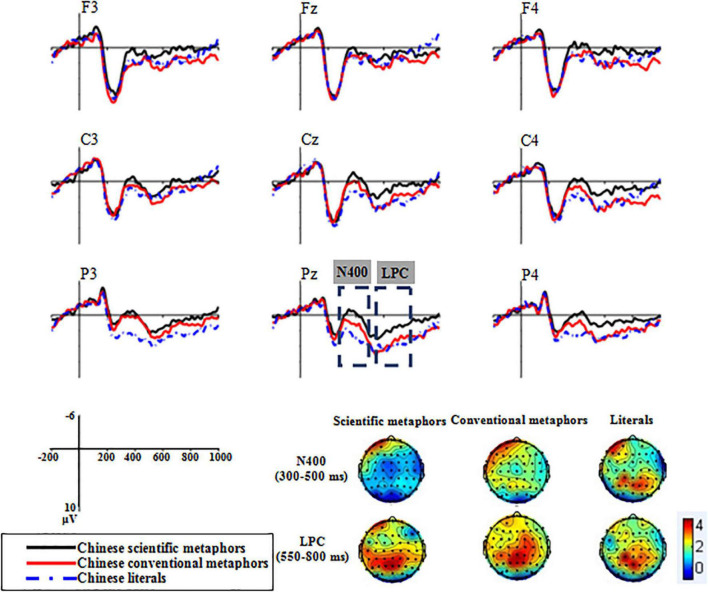
Grand average ERP waveforms of Experiment 1 recorded at the nine chosen electrodes.

According to the Grand average ERP waveforms recorded at the nine chosen electrodes (see Figure 2), there was a sizable negative deflection at about 400 ms, identified as N400, and a later positive deflection appeared from 550 to 800 ms, identified as LPC.

##### 300–500 ms

The condition × region × hemisphere ANOVA revealed a significant main effect of condition [*F*(2,32) = 12.56, *p* < 0.001, *η_*p*_*^2^ = 0.44]. Scientific metaphors elicited the most negative N400 (*M* = 0.61, *SD* = 3.49), followed by conventional metaphors (*M* = 1.75, *SD* = 3.64) and literal sentences (*M* = 2.87, *SD* = 4.63). Pairwise comparisons revealed a significant difference between N400s elicited by scientific metaphors and conventional metaphors [*t*(16) = −2.26, *p* = 0.038]. Meanwhile, both scientific metaphors and conventional metaphors elicited more negative N400s than literal sentences [scientific metaphors: *t*(16) = −6.46, *p* < 0.001; conventional metaphors: *t*(16) = −2.32, *p* = 0.034].

##### 550–800 ms

###### Late Positive Component

The main effect of condition was found to be significant [*F*(2,32) = 5.22, *p* = 0.024, *η_*p*_*^2^ = 0.25]. The lowest LPC amplitude was registered by scientific metaphors (*M* = 1.25, *SD* = 3.94) followed by conventional metaphors (*M* = 3.05, *SD* = 4.26) and literal sentences (*M* = 3.44, *SD* = 4.11). Pairwise comparisons showed that there were significant differences between LPCs elicited by scientific metaphors and conventional metaphors [*t*(16) = −2.33, *p* = 0.033] and by scientific metaphors and literal sentences [*t*(16) = −5.27, *p* < 0.001] but no significant difference between conventional metaphors and literal sentences [*t*(16) = −0.44, *p* = 0.67].

###### Late Negativity

**FIGURE 3:**
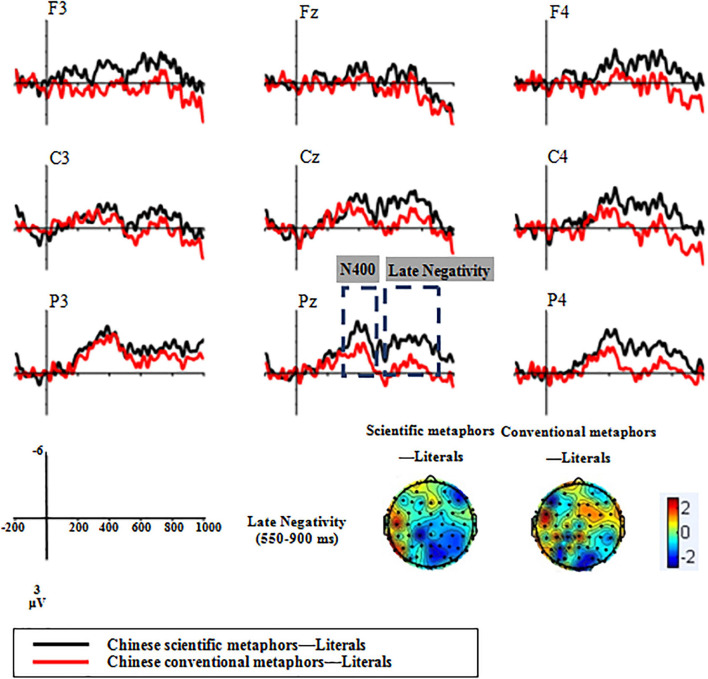
Topographic plots of the two kinds of Chinese metaphors subtracting the literal from the metaphoric in the 300–500 and 550–800 ms time windows.

In addition, the Grand average ERP waveforms of the differences between metaphorical and literal conditions (see Figure 3) showed that both scientific metaphors and conventional metaphors elicited a late negativity in the LPC time window. The 2 × 3 × 3 ANOVA revealed a significant main effect of condition [*F*(1,16) = 5.41, *p* = 0.033, *η_*p*_*^2^ = 0.25]. Scientific metaphors elicited a significantly larger late negativity (*M* = −2.19, *SD* = 3.51) than conventional metaphors (*M* = −0.39, *SD* = 4.48).

### Discussion

Consistent with our predictions, scientific metaphors elicited more negative N400 readings than conventional metaphors. The processing of scientific metaphors involves the conceptual integration between scientific-target and daily-source, while the processing of conventional metaphors involves the conceptual integration of daily-target and daily-source, resulting in the contextual complexity found in scientific metaphors. Compared with conventional metaphors, when processing scientific metaphors, it might be more difficult to search and retrieve stored conceptual knowledge due to the longer distance between the target and source domains (Tang et al., 2017a,b). Meanwhile, larger N400s elicited by scientific metaphors might also indicate the modulation of concreteness. The scientific targets of scientific metaphors were more abstract than the daily targets of conventional metaphors, contributing to the increased negativities found (Forgács et al., 2015).

Consistent with our predictions, both scientific metaphors and conventional metaphors elicited a late negativity partly overlapping in space and time with the LPC (Arzouan et al., 2007a,b; Zucker and Mudrik, 2019). The higher amplitude of scientific metaphors might be caused by the late inference of scientific metaphors from the daily source domain to the scientific target domain in order to understand the related scientific knowledge (Tang et al., 2017a,b). Compared with conventional metaphors, the late stage of scientific metaphor processing involves deeper secondary semantic integration processes. Moreover, as in novel metaphors, the late processing of scientific metaphors is more taxing on working memory (Steinhauer et al., 2010) and more difficult in semantic integration (Zhao et al., 2011; Goldstein et al., 2012; Rutter et al., 2012).

## Experiment 2 (English L2)

### Method

#### Participants

Participants of Experiment 2 were selected from undergraduates with similar ages, language background, and English proficiency as those of Experiment 1. Twenty participants took part in the ERP experiment. As in Experiment 1, the number of subjects included in our final statistical analysis was 17 (8 men, 9 women, average age 21.1 ± 6.54 years).

#### Stimuli

The English stimuli of Experiment 2 were the English counterparts of the Chinese stimuli used in Experiment 1 (see Table 2). The English stimuli followed the “A IS B” structure similar to the structure of the Chinese equivalents. As English is a L2 for the subjects, prior to the neurophysiological study, the English stimuli were tested for familiarity by 40 raters who did not participate in the ERP experiment. Before the test, all raters read a pool of English scientific terms used in the scientific sentences. During the test, the raters were asked to decide whether each expression was familiar or not on a 1–5 scale (1 = not familiar, 5 = highly familiar). A total of 120 English stimuli (40 for each category) with a familiarity higher than 3 were chosen for the ERP experiment. A repeated-measures ANOVA revealed a significant main effect of condition [*F*(2,78) = 39.72, *p* < 0.001, *η_*p*_*^2^ = 0.51]. The familiarity of scientific metaphors was significantly lower than that of conventional metaphors and literal sentences (*p*-values < 0.001), while no significant difference was found between the familiarity of conventional metaphors and literal sentences (*p* = 0.225).

**TABLE 2:** Sample English stimuli.

Scientific metaphors	A charge is flow.
	A conductor is a tunnel.
	A mitochondrion is a code.
	A virus is a killer.
	Sound is wave.
Conventional metaphors	A book is a friend.
	Hangzhou is heaven.
	Language is a bridge.
	Nature is a doctor.
	History is a mirror.
Literal expressions	A professor is a scholar.
	A rose is a plant.
	Beijing is a city.
	Running is a sport.
	Painting is art.

#### Procedure

**FIGURE 4:**
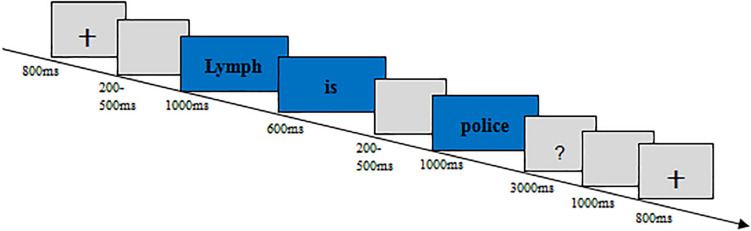
The procedure of Experiment 2.

Same as Experiment 1 (see Figure 4).

#### EEG Recording and Data Analysis

Same as Experiment 1.

### Results Experiment 2

#### Behavioral Performance

Diverging from the result of Experiment 1, the main effect of condition for reaction time was not found to be significant (*p* = 0.48). A repeated-measures ANOVA performed on the accuracy rates yielded significant effects of condition [*F*(2,32) = 14.73, *p* < 0.001, *η_*p*_*^2^ = 0.48]. The difference between the accuracy rates of two metaphorical conditions was not significant (*p* = 0.775), but the accuracy rates of metaphorical conditions were significantly lower than that of the literal condition (*p*-values < 0.001).

#### Electrophysiological Results

**FIGURE 5:**
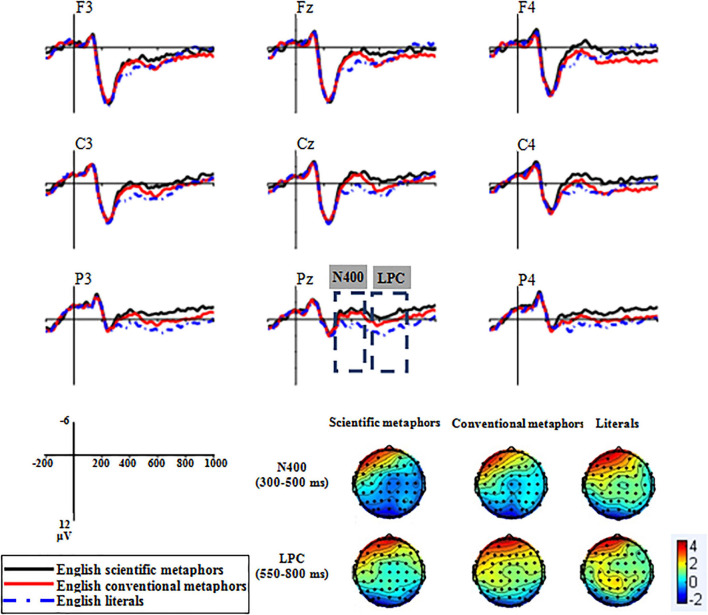
Grand average ERP waveforms of Experiment 2 recorded at the nine chosen electrodes.

According to the Grand average ERP waveforms recorded at the nine chosen electrodes (see Figure 5), there was a sizable negative deflection at about 400 ms (identified as N400) and a later positive deflection from 550 to 800 ms (identified as LPC).

##### 300–500 ms

As with Chinese metaphor processing, the condition × region × hemisphere ANOVA revealed a significant main effect of condition [*F*(2,32) = 20.73, *p* < 0.001, *η_*p*_*^2^ = 0.56]. Scientific metaphors elicited more negative N400s (*M* = −0.02, *SD* = 3.63) than both conventional metaphors (*M* = 0.79, *SD* = 3.58) and literal sentences (*M* = 2.24, *SD* = 2.99). Pairwise comparisons showed a significant difference between N400s elicited by scientific metaphors and conventional metaphors [*t*(16) = −3.07, *p* = 0.007]. Meanwhile, both scientific metaphors and conventional metaphors elicited significantly larger N400s than literal sentences [scientific metaphors: *t*(16) = −5.07, *p* < 0.001; conventional metaphors: *t*(16) = −4.37, *p* < 0.001].

In contrast to Chinese metaphor processing, the differences between English scientific metaphors and conventional metaphors were significant at the frontal [*t*(16) = −2.88, *p* = 0.011], central [*t*(16) = −2.53, *p* = 0.022], and parietal [*t*(16) = −2.85, *p* = 0.012] regions. The difference between N400s elicited by English scientific metaphors and conventional metaphors was marginally significant at the left and middle parietal regions [P3: *t*(16) = −1.79, *p* = 0.091; Pz: *t*(16) = −1.90, *p* = 0.076], significant at the right frontal and central regions [F4: *t*(16) = −3.33, *p* = 0.004; C4: *t*(16) = −3.50, *p* = 0.003], and highly significant at the right parietal region [P4: *t*(16) = −3.65, *p* = 0.002]. Relative to English literal sentences, both English scientific metaphors and English conventional metaphors elicited a more negative N400 at all the three regions {scientific metaphor vs. literal sentences: frontal [*t*(16) = −4.61, *p* < 0.001], central [*t*(16) = −4.21, *p* = 0.001], and parietal [*t*(16) = −6.13, *p* < 0.001] regions; conventional metaphors vs. literal sentences: frontal [*t*(16) = −3.19, *p* = 0.006], central [*t*(16) = −4.17, *p* = 0.001], and parietal [*t*(16) = −5.09, *p* < 0.001] regions}.

##### 550–800 ms

###### Late Positive Component

The condition × region × hemisphere ANOVA revealed a significant main effect of the condition [*F*(2,32) = 5.19, *p* = 0.014, *η_*p*_*^2^ = 0.25]. Scientific metaphors elicited less positive LPCs (*M* = −0.16, *SD* = 3.92) than conventional metaphors (*M* = 1, *SD* = 4.49) and literal sentences (*M* = 1.46, *SD* = 3.42). The condition × region interaction effect was significant [*F*(4,64) = 9.09, *p* < 0.001, *η_*p*_*^2^ = 0.36]. Pairwise comparisons showed that there was a marginally significant difference between LPCs elicited by scientific metaphors and conventional metaphors [*t*(16) = −1.96, *p* = 0.068] and a significant difference between scientific metaphors and literal sentences [*t*(16) = −3.61, *p* = 0.002]. Between conventional metaphors and literal sentences, despite an insignificant main effect of condition [*t*(16) = −0.91, *p* = 0.37], the condition × region interaction effect was significant [*F*(2,32) = 9.78, *p* = 0.001, *η_*p*_*^2^ = 0.38]. *Post-hoc* analysis showed that conventional metaphors elicited significantly lower LPCs at the parietal region [*t*(16) = −2.92, *p* = 0.01].

Since between the two metaphorical conditions, the main effect of condition was significant at the parietal region [*F*(1,16) = 4.59, *p* = 0.048, *η_*p*_*^2^ = 0.22], separate pairwise ANOVAs for the three electrodes at the parietal region (P3, Pz, P4) were performed. It was evident that the LPCs elicited by scientific metaphors were significantly less positive than that of conventional metaphors at P4 [*t*(16) = −2.46, *p* = 0.026] and marginally less significantly positive than that of conventional metaphors at P3 [*t*(16) = −1.83, *p* = 0.085], whereas no such difference was observed at Pz [*t*(16) = −1.62, *p* = 0.125].

###### Late Negativity

**FIGURE 6:**
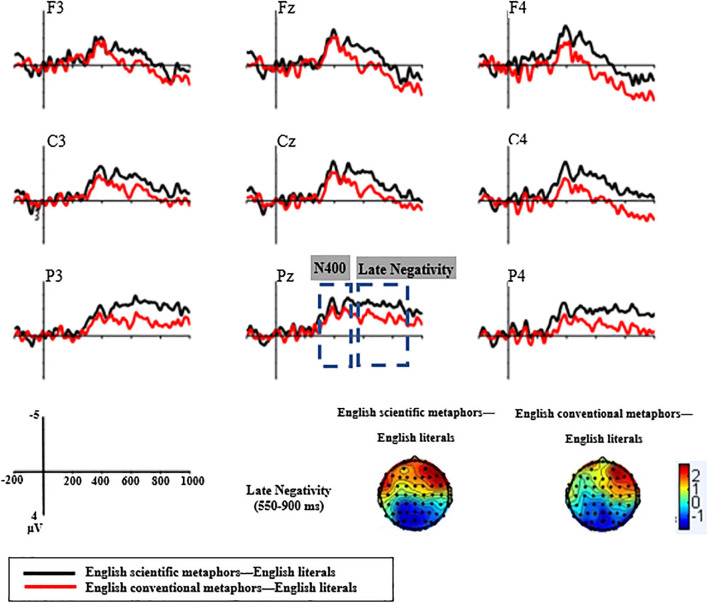
Topographic plots of the two kinds of English metaphors subtracting the literal from the metaphoric in the 300–500 and 550–800 ms time windows.

Analogous to Chinese metaphor processing, English (L2) scientific metaphors and conventional metaphors also elicited a late negativity in the LPC time window (see Figure 6). The 2 × 3 × 3 ANOVA revealed a marginally significant main effect of condition [*F*(1,16) = 3.83, *p* = 0.068, *η_*p*_*^2^ = 0.19]. Scientific metaphors elicited a larger late negativity (*M* = −1.62, *SD* = 2.46) than conventional metaphors (*M* = −0.46, *SD* = 2.68). Follow-up analysis showed that the significant effect was only found at the parietal region [*F*(1,16) = 4.59, *p* = 0.048, *η_*p*_*^2^ = 0.22].

### Comparative Results of Experiments 1 and 2

#### 300–500 ms (N400)

**FIGURE 7:**
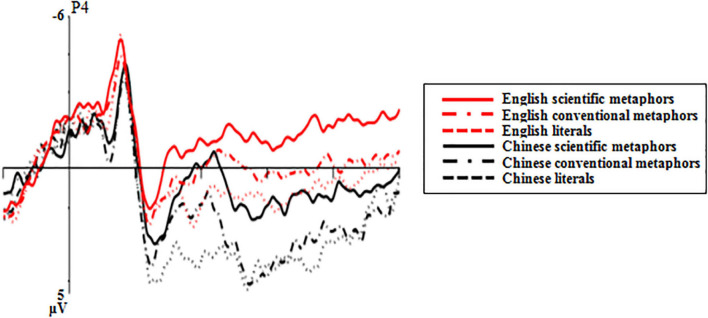
Grand average ERP waveforms of Chinese and English stimuli recorded at the P4 electrode position.

In comparing the results manifested by Chinese and English scientific metaphors (see Figure 7), a condition × region × hemisphere ANOVA showed that the main effect of condition was not significant [*F*(1,16) = 1.79, *p* = 0.46, *η_*p*_*^2^ = 0.04], but there were marginally significant condition × region interactions [*F*(2,32) = 3.37, *p* = 0.073, *η_*p*_*^2^ = 0.17]. Pairwise comparisons showed that English scientific metaphors elicited a significantly larger N400 than Chinese scientific metaphors at the parietal region [*t*(16) = −2.25, *p* = 0.039]. There were no significant effects either between Chinese and English conventional metaphors [*t*(16) = −0.96, *p* = 0.351] nor between Chinese and English literal sentences [*t*(16) = −0.66, *p* = 0.516].

#### 550–800 ms (Late Positive Component)

For the difference between Chinese and English scientific metaphors (see Figure 7), a condition × region × hemisphere ANOVA showed that, despite the insignificant main effect of condition [*F*(1,16) = 1.79, *p* = 0.2, *η_*p*_*^2^ = 0.10], significant condition × region interactions were found [*F*(2,32) = 9.32, *p* = 0.004, *η_*p*_*^2^ = 0.37]. Pairwise comparisons showed that English scientific metaphors elicited a significantly lower LPC than Chinese scientific metaphors at the parietal region [*t*(16) = −3.72, *p* = 0.002], and the difference between the two was highly significant at the right parietal region [P4: *t*(16) = −3.99, *p* = 0.001].

For the difference between Chinese and English conventional metaphors, the condition × region × hemisphere ANOVA revealed a marginally significant main effect of condition [*F*(1,16) = 3.08, *p* = 0.098, *η_*p*_*^2^ = 0.1] and significant condition × region interactions [*F*(2,32) = 6.4, *p* = 0.016, *η_*p*_*^2^ = 0.29]. Pairwise comparisons showed that English conventional metaphors elicited a significantly lower LPC than Chinese conventional metaphors at the parietal region [*t*(16) = −4.97, *p* < 0.001], and the difference between two conditions was highly significant at the right parietal region [P4: *t*(16) = −5.23, *p* < 0.001].

In terms of the differences between Chinese and English literal sentences (see Figure 7), the condition × region × hemisphere ANOVA revealed a marginally significant main effect of condition [*F*(1,16) = 3.54, *p* = 0.078, *η_*p*_*^2^ = 0.18] and significant condition × region interactions [*F*(2,32) = 3.96, *p* = 0.042, *η_*p*_*^2^ = 0.2]. Pairwise comparisons showed that English literal sentences elicited a lower LPC than Chinese literal sentences at the parietal region [*t*(16) = −3.59, *p* = 0.002], and the effect was highly significant at the right parietal region [P4: *t*(16) = −5.36, *p* < 0.001].

### Discussion

Taken together, reduced LPC amplitudes for all three English conditions were maximal over the parietal region, especially over the right parietal region, suggesting that the meanings of both L2 metaphoric and literal sentences are integrated with increased cognitive effort. This result might indicate that late speakers who master two languages asymmetrically are less sensitive to levels of conventionality of metaphoric meanings at the later stage of metaphoric language processing (Jankowiak et al., 2017). Moreover, LPCs are typically considered to reflect the depth of syntactic processing. Compared with L2 processing, the understanding of L1 might involve more syntactic analysis (Van Der Meij et al., 2011).

## General Discussion

The first aim of the current study was to observe brain responses to scientific metaphoric, conventional metaphoric, and literal sentences in Chinese (L1) and English (L2). In line with what was hypothesized, in both the Chinese and English experiments, we observed general between-condition differences, with scientific metaphors eliciting higher N400s than conventional metaphors. However, in the English experiment, more significant effects were found in larger regions over frontal, central, and parietal regions, with a slight right hemisphere bias. Importantly, this finding accords with the behavioral results drawn from the Chinese experiment, which showed lower accuracy rates and longer reaction time for scientific metaphors than with conventional metaphors. This supports the graded salience hypothesis for metaphorical expression. The unique complexity and abstraction of scientific language reduce the explicitness of language expression and have an impact on semantic integration in the later stage of processing. The longer distance between the scientific target domain and the daily source domain for scientific metaphor comprehension might increase cognitive effort in conceptual integration (Tang et al., 2017a,b). According to the Career of Metaphor Model (Bowdle and Gentner, 2005), the processing mechanism of metaphors is regulated by their familiarity. The understanding of metaphors with low familiarity requires the construction of metaphorical meaning through lexical semantic processes, while the understanding of metaphors with high familiarity is mainly carried out through semantic retrieval (Coulson and Van Petten, 2002; Lai and Curran, 2013). In addition, compared with literal expressions, conventional metaphors elicited a larger N400 response, which might indicate that despite being frequently used with high familiarity, conventional metaphors require more resource-intensive mappings between concepts than literal expressions.

In both Chinese and English experiments, LPC amplitudes evoked by scientific metaphors were smaller than those elicited by conventional metaphors and literal sentences. Lower LPC amplitudes for scientific metaphors might be caused by a sustained negativity, indexing the integration process of conceptually taxing meanings. Another explanation could be due to reprocessing operations after an initial failure in meaning interpretation (Ruchkin et al., 1988; Rataj, 2014) or alternatively extra working memory load for complex semantic processing (Ruchkin et al., 1988; Anderson et al., 1996; Oberauer et al., 2001; Jiang et al., 2009). According to some monolingual research on metaphors, novel metaphors elicited larger late negativity amplitudes than conventional metaphors, which was interpreted as the continuation of information retrieval or access to the non-literal route when understanding novel metaphors (Arzouan et al., 2007a), and as the ongoing difficulty of meaning integration indexed as the continuation of the N400 effect (Rutter et al., 2012). The larger late negativity of scientific metaphors might further indicate more difficult reintegration of the two domains, especially when the knowledge inference is involved at the later stage of scientific metaphor processing.

Second, the current study aimed to observe whether language nativeness modulates meaning integration mechanisms of scientific metaphors, to which the N400 response is sensitive. More specifically speaking, we were interested in whether L2 scientific metaphors would elicit similar N400s as L1 equivalents for late unbalanced bilingual speakers.

Between-language effects were found with English scientific metaphors eliciting more negative N400s than Chinese ones at the parietal region. Such enhanced N400 response to L2 relative to L1 stimuli is contrary to the results of some bilingual research (Proverbio et al., 2002; Moreno et al., 2008; Midgley et al., 2009; Newman et al., 2012; Heidlmayr et al., 2015). Within the memory system, weaker semantic interconnectivity for L2 compared with L1 words might explain the increased N400 amplitude (Midgley et al., 2009). From the opposite perspective, within the semantic network, larger interconnectivity for L1 words might be linked to the N400 L1/L2 effect (increased N400 amplitudes for L1 relative to L2 words). However, for scientific words, the picture might be different. Compared with conventional words, the semantic connectivity of scientific words might be quite weak in both native and non-native languages. This conjecture would seem to be supported by the behavioral results of the present English experiment. Such weak interconnectivity for scientific words might evoke similar activity in long-term memory in both languages for information retrieval. Unlike the daily words used in conventional metaphors, the scientific terms used in scientific metaphors are even less frequently used, which might result in the much lower familiarity for L2 relative to L1 scientific words. Therefore, the semantic processing of second language scientific terms is more challenging than that of Chinese ones. Moreover, for ordinary L2 speakers (Chang and Wang, 2016), the processing of L2 vocabulary often requires a greater degree of suppression of native vocabulary (Heidlmayr et al., 2015; Wu and Thierry, 2017), which further leads to difficulty in processing L2 words, especially scientific terms.

In addition, at the frontal, central, and parietal regions of the right hemisphere, English scientific metaphors elicited higher N400s than English conventional metaphors, indicating the special role of the right hemisphere in second language processing (Van Der Meij et al., 2011), which supports the Fine-Coarse Semantic Coding Theory (Mashal et al., 2015). Meanwhile, at the left parietal region, there was a marginally significant difference between the two metaphorical sentences, probably showing that the left hemisphere is also involved in understanding L2 scientific metaphors (Kim et al., 2017; Segal and Gollan, 2018).

Third, the current study aimed to observe the modulation of language nativeness for cognitive mechanisms involved at the later stage of meaning reintegration, which the LPC is sensitive to. In line with our predictions, within the LPC time frame, at the parietal region, we observed between-language differences with a slight right hemisphere bias, with smaller LPC responses evoked by the three English conditions than their Chinese counterparts. Reduced LPC amplitudes for scientific and conventional metaphors in the non-native language suggest more demanding cognitive effort to integrate the meaning of both novel and familiar metaphors in an L2 context. This result might indicate that late speakers who master two languages asymmetrically are less sensitive to the levels of conventionality of metaphoric meanings at the later stage of metaphoric language processing.

Inconsistent with the processing of Chinese scientific metaphors, English scientific metaphors only elicited lower LPCs than English conventional metaphors at the parietal region. That is to say, the LPC distribution of L1 scientific metaphors covered a larger area than that of L2 scientific metaphors (Jankowiak et al., 2017; Segal and Gollan, 2018), probably because it might be quite difficult for late L2 learners to reach a similar processing depth as displayed by the native speakers (Newman et al., 2012). In addition, both the left and right hemispheres are involved in the processing of L2 scientific metaphors (Chen et al., 2013), and the right parietal region might play a particularly important role.

## Conclusion

Through a comparative analysis of the ERP components elicited by scientific metaphors in English and Chinese, this study examined brain responses to scientific metaphors in L2. It was found that nativeness modulates the cognitive cost for semantic integration at an early stage and for semantic reintegration and knowledge inference at a later period, supporting the Career of Metaphor Model and the Graded Salience Hypothesis.

In addition, the scalp distributions of the N400s and the late component elicited by scientific metaphors in L2 reinforce the essential role of the parietal region (especially the right parietal region) in processing L2, supporting the Fine-Coarse Semantic Coding Theory. More abundant types of stimuli could be added to subsequent follow-up experiments for more comparative analysis so as to continuously verify and improve existing studies.

## Data Availability Statement

The raw data supporting the conclusions of this article will be made available by the authors, without undue reservation.

## Ethics Statement

The studies involving human participants were reviewed and approved by Ethics Committee in Shaanxi Normal University. The patients/participants provided their written informed consent to participate in this study.

## Author Contributions

XT and WR contributed to conception and design of the study. XT, MH, YH, and SH performed the data collection. LS, MH, and PY performed the analysis. XT wrote the first draft of the manuscript. All authors contributed to manuscript revision, read, and approved the submitted version.

## Conflict of Interest

The authors declare that the research was conducted in the absence of any commercial or financial relationships that could be construed as a potential conflict of interest.

## Publisher’s Note

All claims expressed in this article are solely those of the authors and do not necessarily represent those of their affiliated organizations, or those of the publisher, the editors and the reviewers. Any product that may be evaluated in this article, or claim that may be made by its manufacturer, is not guaranteed or endorsed by the publisher.

## References

[B1] Anderson J. R. Reder L. M. Lebiere C. (1996). Working memory: activation limitations on retrieval. *Cogn. Psychol.* 30 221–256. 10.1006/cogp.1996.0007 8660785

[B2] Arzouan Y. Goldstein A. Faust M. (2007a). Brainwaves are stethoscopes: ERP correlates of novel metaphor comprehension. *Brain Res.* 1160 69–81. 10.1016/j.brainres.2007.05.034 17597591

[B3] Arzouan Y. Goldstein A. Faust M. (2007b). Dynamics of hemispheric activity during metaphor comprehension: electrophysiological measures. *Neuroimage* 36 222–231. 10.1016/j.neuroimage.2007.02.015 17428685

[B4] Bowdle B. F. Gentner D. (2005). The career of metaphor. *Psychol. Rev.* 112 193–216. 10.1037/0033-295X.112.1.193 15631593

[B5] Braunstein V. Ischebeck A. Brunner C. Grabner R. H. Stamenov M. Neuper C. (2012). Investigating the influence of proficiency on semantic processing in bilinguals: an ERP and ERD/S analysis. *Acta Neurobiol. Exp.* 72 421–438. 23377272 10.55782/ane-2012-1913

[B6] Brouwer H. Fitz H. Hoeks J. (2012). Getting real about semantic illusions: rethinking the functional role of the P600 in language comprehension. *Brain Res.* 1446 127–143. 10.1016/j.brainres.2012.01.055 22361114

[B7] Chang X. Wang P. (2016). Influence of second language proficiency and syntactic structure similarities on the sensitivity and processing of english passive sentence in late chinese-english bilinguists: an ERP study. *J. Psychol. Res.* 45 85–101. 10.1007/s10936-014-9319-1 25304973

[B8] Chen H. Peng X. Zhao Y. (2013). An ERP study on metaphor comprehension in the bilingual brain. *Chin. J. Appl. Ling.* 36 505–519. 10.1515/cjal-2013-0034

[B9] Coulson S. Van Petten C. (2002). Conceptual integration and metaphor: an event-related potential study. *Memory Cogn.* 30 958–968. 10.3758/BF03195780 12450098

[B10] Coulson S. Van Petten C. (2007). A special role for the right hemisphere in metaphor comprehension? ERP evidence from hemifield presentation. *Brain Res.* 1146 128–145. 10.1016/j.brainres.2007.03.008 17433892

[B11] De Grauwe S. Swain A. Holcomb P. J. Ditman T. Kuperberg G. R. (2010). Electrophysiological insights into the processing of nominal metaphors. *Neuropsychologia* 48 1965–1984. 10.1016/j.neuropsychologia.2010.03.017 20307557 PMC2907657

[B12] Forgács B. Bardolph M. Amsel B. D. DeLong K. A. Kutas M. (2015). Metaphors are physical and abstract: ERPs to metaphorically modified nouns resemble ERPs to abstract language. *Front. Hum. Neurosci.* 9:28. 10.3389/fnhum.2015.00028 25713520 PMC4322728

[B13] Forgács B. Lukács Á Pléh C. (2014). Lateralized processing of novel metaphors: disentangling figurativeness and novelty. *Neuropsychologia* 56 101–109. 10.1016/j.neuropsychologia.2014.01.003 24418155

[B14] Goldstein A. Arzouan Y. Faust M. (2012). Killing a novel metaphor and reviving a dead one: ERP correlates of metaphor conventionalization. *Brain Lang.* 123 137–142. 10.1016/j.bandl.2012.09.008 23063676

[B15] Gratton G. Coles M. G. Donchin E. (1983). A new method for off-line removal of ocular artifact. *Electroencephalogr. clin. neurophysiol.* 55, 468–484. 10.1016/0013-4694(83)90135-9 6187540

[B16] Heidlmayr K. Hemforth B. Moutier S. Isel F. (2015). Neurodynamics of executive control processes in bilinguals: evidence from ERP and source reconstruction analyses. *Front. Psychol.* 6:1–17. 10.3389/fpsyg.2015.00821 26124740 PMC4467069

[B17] Jankowiak K. Rataj K. Naskrecki R. (2017). To electrify bilingualism: electrophysiological insights into bilingual metaphor comprehension. *PLoS One* 12:1–30. 10.1371/journal.pone.0175578 28414742 PMC5393611

[B18] Jiang X. Tan Y. Zhou X. (2009). Processing the universal quantifier during sentence comprehension: ERP evidence. *Neuropsychologia* 47 1799–1815. 10.1016/j.neuropsychologia.2009.02.020 19428412

[B19] Kaan E. Harris A. Gibson E. Holcomb P. J. (2000). The P600 as an index of syntactic integration difficulty. *Lang. Cogn. Proc.* 15 159–201. 10.1080/016909600386084

[B20] Kim S. Y. Liu L. Cao F. (2017). How does first language (L1) influence second language (L2) reading in the brain? Evidence from korean-english and chinese-english bilinguals. *Brain Lang.* 171 1–13. 10.1016/j.bandl.2017.04.003 28437658

[B21] Kotz S. A. Rothermich K. Schmidt-Kassow M. (2012). “Sentence comprehension in healthy and braindamaged populations,” in *The Handbook of the Neuropsychology of Language* ed. Faust M. (Malden: Blackwell Publishing), 760–777.

[B22] Kutas M. Federmeier K. D. (2000). Electrophysiology reveals semantic memory use in language comprehension. *Trends Cogn. Sci.* 4 463–470. 10.1016/S1364-6613(00)01560-6 11115760

[B23] Kutas M. Hillyard S. A. (1980). Reading senseless sentences: brain potentials reflect semantic incongruity. *Science* 207 203–205. 10.1126/science.7350657 7350657

[B24] Lai V. T. Curran T. (2013). ERP evidence for conceptual mappings and comparison processes during the comprehension of conventional and novel metaphors. *Brain Lang.* 127 484–496. 10.1016/j.bandl.2013.09.010 24182839

[B25] Lai V. T. Curran T. Menn L. (2009). Comprehending conventional and novel metaphors: AN ERP study. *Brain Res.* 1284 145–155. 10.1016/j.brainres.2009.05.088 19505446

[B26] Lakoff G. Johnson M. (1980). *Metaphors We Live By.* Chicago: University of Chicago Press.

[B27] Lopez-Calderon J. Luck S. J. (2014). ERPLAB: an open-source toolbox for the analysis of event-related potentials. *Front. Hum. Neurosci.* 8, 213. 10.3389/fnhum.2014.00213 24782741 PMC3995046

[B28] Ma Q. Hu L. Xiao C. Bian J. Jin J. Wang Q. (2016). Neural correlates of multimodal metaphor comprehension: evidence from event-related potentials and time-frequency decompositions. *Int. J. Psychophysiol.* 109 81–91. 10.1016/j.ijpsycho.2016.09.007 27622382

[B29] Mashal N. Borodkin K. Maliniak O. Faust M. (2015). Hemispheric involvement in native and non-native comprehension of conventional metaphors. *J. Neurolinguist.* 35 96–108. 10.1016/j.jneuroling.2015.04.001

[B30] Midgley K. J. Holcomb P. J. Grainger J. (2009). Language effects in second language learners and proficient bilinguals investigated with event-related potentials. *J. Neurolinguist.* 22 281–300. 10.1016/j.jneuroling.2008.08.001 19430590 PMC2678859

[B31] Moreno E. M. Fornells A. R. Laine M. (2008). Event-related potentials (ERPs) in the study of bilingual language processing. *J. Neurolinguist.* 21 477–508. 10.1016/j.jneuroling.2008.01.003

[B32] Moreno E. M. Kutas M. (2005). Processing semantic anomalies in two languages: an electrophysiological exploration in both languages of Spanish–english bilinguals. *Cogn. Brain Res.* 22 205–220. 10.1016/j.cogbrainres.2004.08.010 15653294

[B33] Newman A. J. Tremblay A. Nichols E. S. Neville H. J. Ullman M. T. (2012). The influence of language proficiency on lexical semantic processing in native and late learners of english. *J. Cogn. Neurosci.* 24 1205–1223. 10.1162/jocn_a_00143 21981676 PMC4447492

[B34] Oberauer K. Demmrich A. Mayr U. Kliegl R. (2001). Dissociating retention and access in working memory: an age-comparative study of mental arithmetic. *Memory Cogn.* 29 18–33. 10.3758/BF03195737 11277461

[B35] Phillips N. A. Segalowitz N. O’Brien I. Yamasaki N. (2004). Semantic priming in a first and second language: evidence from reaction time variability and event-related brain potentials. *J. Neurolinguist.* 17 237–262. 10.1016/s0911-6044(03)00055-1

[B36] Proverbio A. M. Cok B. Zani A. (2002). Electrophysiological measures of language processing in bilinguals. *J. Cogn. Neurosci.* 14 994–1017. 10.1162/089892902320474463 12419124

[B37] Pynte J. Besson M. Robichon F. H. Poli J. (1996). The time-course of metaphor comprehension: an event-related potential study. *Brain Lang.* 55 293–316. 10.1006/brln.1996.0107 8954602

[B38] Rataj K. (2014). Surfing the brainwaves of metaphor comprehension. *Poznan Stud. Contemp. Ling.* 50 55–73. 10.1515/psicl-2014-0004

[B39] Ruchkin D. S. Mahaffey D. Sutton S. (1988). Toward a functional categorization of slow waves. *Psychophysiology* 25 339–353. 10.1111/j.1469-8986.1988.tb01253.x 3406333

[B40] Rutter B. Kröger S. Hill H. Windmann S. Hermann C. Abraham A. (2012). Can clouds dance? Part 2: an ERP investigation of passive conceptual expansion. *Brain Cogn.* 80 301–310. 10.1016/j.bandc.2012.08.003 23137771

[B41] Segal D. Gollan T. H. (2018). What’s left for balanced bilinguals? Language proficiency and item familiarity affect left-hemisphere specialization in metaphor processing. *Neuropsychology* 32 866–879. 10.1037/neu0000467 30160502 PMC6217862

[B42] Steinhauer K. Drury J. E. Portner P. Walenski M. Ullman M. T. (2010). Syntax, concepts, and logic in the temporal dynamics of language comprehension: Evidence from event-related potentials. *Neuropsychologia* 48 1525–1542. 10.1016/j.neuropsychologia.2010.01.013 20138065 PMC2862874

[B43] Tang X. Qi S. Jia X. Wang B. Ren W. (2017a). Comprehension of scientific metaphors: complementary processes revealed by ERP. *J. Neurolinguist.* 42 12–22. 10.1016/j.jneuroling.2016.11.003

[B44] Tang X. Qi S. Wang B. Jia X. Ren W. (2017b). The temporal dynamics underlying the comprehension of scientific metaphors and poetic metaphors. *Brain Res.* 1655 33–40. 10.1016/j.brainres.2016.11.005 27845031

[B45] Tartter V. C. Gomes H. Dubrovsky B. Molholm S. Stewart R. V. (2002). Novel metaphors appear anomalous at least momentarily: evidence from N400. *Brain Lang.* 80 488–509. 10.1006/brln.2001.2610 11896654

[B46] Van Der Meij M. Cuetos F. Carreiras M. Barber H. A. (2011). Electrophysiological correlates of language switching in second language learners. *Psychophysiology* 48 44–54. 10.1111/j.1469-8986.2010.01039.x 21143487

[B47] van Herten M. Kolk H. H. J. Chwilla D. J. (2005). An ERP study of P600 effects elicited by semantic anomalies. *Cogn. Brain Res.* 22 241–255. 10.1016/j.cogbrainres.2004.09.002 15653297

[B48] Weber-Fox C. M. Neville H. J. (1996). Maturational constraints on functional specializations for language processing: ERP and behavioral evidence in bilingual speakers. *J. Cogn. Neurosci.* 8, 231–256. 10.1162/jocn.1996.8.3.231 23968150

[B49] Weiland H. Bambini V. Schumacher P. B. (2014). The role of literal meaning in figurative language comprehension: evidence from masked priming ERP. *Front. Hum. Neurosci.* 8:1–17. 10.3389/fnhum.2014.00583 25136309 PMC4120764

[B50] Wu Y. J. Thierry G. (2017). Brain potentials predict language selection before speech onset in bilinguals. *Brain Lang.* 171 23–30. 10.1016/j.bandl.2017.04.002 28445784

[B51] Zhao M. Meng H. Xu Z. Du F. Liu T. Li Y. (2011). The neuromechanism underlying verbal analogical reasoning of metaphorical relations: an event-related potentials study. *Brain Res.* 1425 62–74. 10.1016/j.brainres.2011.09.041 22018690

[B52] Zucker L. Mudrik L. (2019). Understanding associative vs. abstract pictorial relations: an ERP study. *Neuropsychologia* 133 1–16. 10.1016/j.neuropsychologia.2019.107127 31279832

